# Effect of methylglyoxal on multidrug-resistant *Pseudomonas aeruginosa*

**DOI:** 10.3389/fmicb.2014.00180

**Published:** 2014-04-17

**Authors:** Katsuhiko Hayashi, Aiko Fukushima, Mitsuko Hayashi-Nishino, Kunihiko Nishino

**Affiliations:** ^1^Laboratory of Microbiology and Infectious Diseases, Division of Special Projects, Institute of Scientific and Industrial Research, Osaka UniversityIbaraki, Osaka, Japan; ^2^Graduate School of Pharmaceutical Sciences, Osaka UniversitySuita, Japan

**Keywords:** manuka honey, methylglyoxal, drug efflux system, multidrug resistance, *Pseudomonas aeruginosa*

## Abstract

Honey has a complex chemistry, and its broad-spectrum antimicrobial activity varies with floral source, climate, and harvesting conditions. Methylglyoxal was identified as the dominant antibacterial component of manuka honey. Although it has been known that methylglyoxal has antibacterial activity against gram-positive bacteria, including methicillin-resistant *Staphylococcus aureus* and vancomycin-resistant *Enterococcus*, there is not much information describing its activity against gram-negative bacteria. In this study, we report the effect of methylglyoxal against multidrug-resistant *Pseudomonas aeruginosa* (MDRP) using 53 clinically isolated strains. We also assessed the effect of deleting the five multidrug efflux systems in *P. aeruginosa*, as well as the efflux systems in *Escherichia coli* and *Salmonella enterica* serovar Typhimurium, on MICs of methylglyoxal. Our results indicate that methylglyoxal inhibits the growth of MDRP at concentrations of 128–512 μg/ml (1.7–7.1 mM) and is not recognized by drug efflux systems.

## Introduction

*Pseudomonas aeruginosa* is endemic among critically ill patients, and multidrug-resistant strains are increasingly being isolated in intensive care units (Ortega et al., 2004). Because *P. aeruginosa* is a virulent organism susceptible to a limited number of antibiotic agents, infections caused by this organism are difficult to cure and often require combination therapy. Multidrug-resistant *P. aeruginosa* (MDRP) has been defined as *P. aeruginosa* resistant to imipenem, amikacin, and ciprofloxacin (Sekiguchi et al., 2007). The increasing resistance of *P. aeruginosa* is a growing threat to the clinical management of such infections (Ortega et al., 2004).

In bacteria, resistance to bactericidal agents is often associated with multidrug efflux systems, which decrease cellular drug accumulation (Nikaido, 1996). In gram-negative bacteria, systems belonging to the resistance/nodulation/division (RND) family are particularly effective in generating resistance because they form a tripartite complex with the periplasmic proteins of the membrane fusion protein family and an outer membrane channel, ensuring that drugs are pumped out directly to the external medium (Nikaido and Pages, 2012). *P. aeruginosa* expresses several RND-type multidrug efflux systems, including MexAB-OprM, MexCD-OprJ, MexEF-OprN, and MexXY, which are significant determinants of multidrug resistance in laboratory and clinical isolates (Poole, 2004; Piddock, 2006; Lister et al., 2009). These systems are three-component systems comprising antiporters of the RND family driven by proton motive force (MexB, MexD, MexF, and MexY), outer membrane channels (OprM, OprJ, and OprN), and periplasmic membrane fusion proteins (MexA, MexC, MexF, and MexX). These pumps function in a manner similar to AcrAB–TolC, which is the best-studied RND-type multidrug pump of *Escherichia coli* (Nakashima et al., 2011; Nikaido, 2011). It is necessary to develop drugs that are not recognized by the efflux pumps to prevent multidrug resistance modulated by drug efflux systems.

Honey has several antibacterial features that are distinct from classical antibiotics, including high osmolarity, low pH, and generation of hydrogen peroxide by the bee-derived enzyme glucose oxidase (Allen et al., 1991). Antibacterial phenolic components have been identified in honey (Weston et al., 1999), and an antimicrobial peptide has been discovered in a Dutch medical-grade honey produced from an undisclosed floral source cultivated in greenhouses (Kwakman et al., 2010). Manuka honey is derived from nectar that has been collected by honey bees (*Apis mellifera*) foraging on a shrub known as manuka (*Leptospermum scoparium*) that is indigenous to New Zealand. Manuka honey is broad in spectrum, able to inhibit a diverse range of bacterial and yeast pathogens, and equally effective against multidrug-resistant bacteria (Blair et al., 2009; Henriques et al., 2010; Kwakman et al., 2011). It is used in modern wound-care formulations and has been shown to eradicate methicillin-resistant *Staphylococcus aureus* (MRSA) from wounds (Natarajan et al., 2001; Blaser et al., 2007; Gethin and Cowman, 2008; Visavadia et al., 2008). Clinically isolated strains of methicillin-susceptible and -resistant staphylococci were shown to be equally susceptible to manuka honey *in vitro*, with minimum inhibitory concentrations (MICs) reported to be <3% (v/v) [equivalent to 41,000 mg/L or 4.1% (w/v)] (Cooper et al., 1999, 2002b). Methylglyoxal was identified as the dominant active antibacterial component of manuka honey (Mavric et al., 2008; Adams et al., 2009b). Active manuka honey contains high levels of the reactive dicarbonyl methylglyoxal (Mavric et al., 2008; Adams et al., 2009a), which is formed nonenzymatically from nectar-derived dihydroxyacetone during ripening. Methylglyoxal was also found to be produced from dihydrocyacetone phosphate in *E. coli*, initiating a bypass of the glycolytic pathway (Cooper and Anderson, 1970). It was suggested that methylglyoxal inhibits protein synthesis by reacting with guanine residues in RNA and its precursors. It also inhibits DNA synthesis by reacting with guanine residues in DNA and its precursors (Krymkiewicz et al., 1971).

It has been known that methylglyoxal has antibacterial activity against gram-positive bacteria, including MRSA and vancomycin-resistant *Enterococcus*. It was also reported that methylglyoxal containing manuka honey is biocidal against *S. aureus* strains at a concentration of 33–66% w/v (equivalent methylglyoxal concentration, 260–530 μg/ml) (Jervis-Bardy et al., 2011). However, there is not much information describing methylglyoxal activity against gram-negative bacteria. Although it was previously reported that manuka honey is bactericidal against *P. aeruginosa* (Roberts et al., 2012), the effect of methylglyoxal on MDRP has been unknown. In this study, we report the antibacterial effect of methylglyoxal on MDRP using 53 clinically isolated strains. We also demonstrate that methylglyoxal is not recognized by drug efflux systems in *P. aeruginosa*, *Salmonella enterica*, and *E. coli*.

## Materials and methods

### Bacterial strains and growth conditions

The bacterial strains used in this study are listed in Table 1. We used *P. aeruginosa* PAO1 (Stover et al., 2000), *S. enterica* serovar Typhimurium ATCC14028s (Fields et al., 1986), and *E. coli* MG1655 (Blattner et al., 1997) as wild-type strains. All clinically isolated MDRP strains, which showed resistance to imipenem, amikacin, and ciprofloxacin, were kindly provided by Biomedical Laboratories, Inc. (Tokyo, Japan).

**Table 1:** **Bacterial strains used in this study**.

**Strain**	**Characteristics**	**Source or references**
MDRP1, 2, 4, 5, 7, 8, 9, 10, 12, 13, 14, 19, 20, 21, 24, 25, 29, 30, 31, 32, 33, 38, 39, 41, 42, 44, 45, 46, 50, 57, 60, 62, 63, 67, 71, 72, 74, 75, 83, 86, 87, 88, 92, 93, 94, 95, 96, 98, 100, 101, 103, 105, 106	MDRP strains, clinically isolated	Biomedical Laboratories, Inc.
PAO1	*Pseudomonas aeruginosa* wild-type	Stover et al., 2000
PMX52	Δ*mexAB oprM mexCD oprJ mexEF oprN mexXY mexHI opmD*, PAO1 derivative	Sekiya et al., 2003
MG1655	*Escherichia coli* wild-type	Blattner et al., 1997
NKE1329	Δ*acrB acrD mdtABC mdtEF acrEF*, MG1655 derivative	This study
NKE95	Δ*tolC*, MG1655 derivative	This study
ATCC14028s	*Salmonella enterica* serovar Typhimurium wild-type	Fields et al., 1986
NKS196	Δ*acrAB acrEF acrD mdtABC mdsABC emrAB mdfA mdtK macAB*, ATCC14028s derivative	Horiyama et al., 2011
NKS233	Δ*tolC*, ATCC14028s derivative	Yamasaki et al., 2011

MDRP, multidrug-resistant Pseudomonas aeruginosa.

### Construction of gene deletion mutants

*P. aeruginosa* PMX52 (Sekiya et al., 2003), a PAO1-derived strain lacking the genes encoding the MexAB-OprM, MexCD-OprJ, MexEF-OprN, MexXY, and MexHI-OpmD drug efflux systems, was kindly provided by Tomofusa Tsuchiya of Ritsumeikan University, Japan. *S. enterica* serovar Typhimurium strains NKS196 (Δ*acrAB acrEF acrD mdtABC mdsABC emrAB mdfA mdtK macAB*) and NKS233 (Δ*tolC*) were constructed as described previously (Horiyama et al., 2011; Yamasaki et al., 2011).

To construct *E. coli* strains NKE1329 (Δ*acrB acrD mdtABC mdtEF acrEF*) and NKE95 (Δ*tolC*), we performed gene disruption using procedures described previously (Datsenko and Wanner, 2000). The following oligonucleotide primers were used for the construction of the mutants: *acrB*-P1 (AAAAAGGCCGCTTACGCGGCCTTAGTGATTACACGTTGTAGTGTAGGCTGGAGCTGCTTC); *acrB*-P2 (GAACAGTCCAAGTCTTAACTTAAACAGGAGCCGTTAAGACCATATGAATATCCTCCTTAG); *acrD*-P1 (TGAAAAAGGCGACACATTGGCATGTCGCCTTTTTTATTGCGTGTAGGCTGGAGCTGCTTC); *acrD*-P2 (AAGCCTACAACGATACGCAGAAACACGAGGTCCTCTTTTACATATGAATATCCTCCTTAG); *mdtA*-P1 (ATCATTCCGCGAAACGTTTCAGGAAGAGAAACTCTTAACGGTGTAGGCTGGAGCTGCTTC); *mdtC*-P2 (GAGATACACCACCGGCGTGGTATACAGCGTAAGGAGCTGGCATATGAATATCCTCCTTAG); *mdtE*-P1 (TTAAAGAACCGTTATTTCTCAAGAATTTTCAGGGACTAAAGTGTAGGCTGGAGCTGCTTC); *mdtF*-P2 (AGGCTGAACCTTCATGTTCAACCTTACTCTCATTTACACGCATATGAATATCCTCCTTAG); *acrE*-P1 (TTGGGTAAATAACGCGCTTTTGGTTTTTTGAGGAATAGTAGTGTAGGCTGGAGCTGCTTC); *acrF*-P2 (AAATAATAAAGGCACCCGAAAGCGCCTTTATGTTTCTGATCATATGAATATCCTCCTTAG); *tolC*-P1 (ACTGGTGCCGGGCTATCAGGCGCATAACCATCAGCAATAGGTGTAGGCTGGAGCTGCTTC); and *tolC*-P2 (TTACAGTTTGATCGCGCTAAATACTGCTTCACCACAAGGACATATGAATATCCTCCTTAG). The chloramphenicol resistance gene *cat* or the kanamycin resistance gene *aph*, flanked by Flp recognition sites, was amplified by PCR using the primers listed above. The resulting PCR products were used to transform *E. coli* MG1655, which harbors plasmid pKD46, expressing Red recombinase. The chromosomal structures of the mutated *loci* were verified by PCR; *cat* and *aph* were further eliminated using the plasmid pCP20, as described previously (Datsenko and Wanner, 2000). To construct the NKE1329 strain, the deletions were transferred to strains by P22 transduction, as described previously Davis et al. (1980).

### Determination of MICs of antimicrobial compounds

Antibacterial activities were determined on Muller Hinton II agar (Becton Dickinson & Co., Franklin Lakes, NJ, USA) plates containing methylglyoxal (32–2048 μg/ml), imipenem (0.0625–2048 μg/ml), amikacin (0.125–4096 μg/ml), or ciprofloxacin (0.0078–2048 μg/ml) (Sigma, St. Louis, MO, USA). Agar plates were prepared using the two-fold agar dilution technique. Bacteria were grown at 37°C overnight and then tested at a final inoculum volume of 1 × 10^5^ cfu/μl using a multipoint inoculator (Sakuma Seisakusyo, Tokyo, Japan). The inoculated agar plates were examined after incubation at 37°C for 16 h. MIC was the lowest concentration of a compound that inhibited cell growth.

### Measurement of bacterial growth in the presence of methylglyoxal

*E. coli* (MG1655, NKE1329, and NKE95) and *S. enterica* (ATCC14028s, NKS196, and NKS233) strains were grown in Luria–Bertani broth (Becton Dickinson & Co., Franklin Lakes, NJ, USA), and *P. aeruginosa* (PAO1 and PMX52) strains were grown in Muller Hinton II (MHII) broth (Becton Dickinson & Co., Franklin Lakes, NJ, USA). Bacterial cells were cultured overnight at 37°C, and then 100 μl of cell cultures were diluted in 5 ml of the same medium. The diluted bacterial cells were incubated at 37°C until OD_600_ reached 0.5. Then, the bacterial cells were diluted in the same medium to an OD_600_ of 0.05. This diluted bacterial cells were incubated in NUNC Edge 96-well plates (Thermo scientific, MA, USA) with shaking at 37°C for 7 h. Bacterial growth was monitored using an Infinite M200 Pro plate reader (Tecan, Männedorf, Switzerland).

## Results and discussion

### MICs of imipenem, amikacin, or ciprofloxacin for clinically isolated MDRP

MDRP has been defined as *P. aeruginosa* resistant to imipenem (MIC, ≥16 μg/ml), amikacin (≥32 μg/ml), and ciprofloxacin (≥4 μg/ml) (Sekiguchi et al., 2007). Using this criterion, we determined that all 53 clinical isolates were MDRP (Table 2). The highest MIC of imipenem for strains MDRP7, 14, 45, 74, and 83 was 512 μg/ml. The highest MIC of amikacin for strains MDRP30 and 57 was 2048 μg/ml. The highest MIC of ciprofloxacin for strains MDRP10, 29, and 32 was 1024 μg/ml.

**Table 2:** **Susceptibility of MDRP strains to antimicrobial compounds**.

	**MIC (μg/ml)**			
**Strain**	**MGO**	**IPM**	**AMK**	**CPFX**
MDRP1, 31, 75, 100	512	128	1024	64
MDRP2	512	32	128	32
MDRP4	256	128	1024	64
MDRP5	256	128	128	32
MDRP7	512	512	512	64
MDRP8	512	256	512	64
MDRP9, 19, 86, 93	512	256	1024	64
MDRP10, 29	512	256	1024	1024
MDRP12	512	16	256	64
MDRP13	512	32	256	128
MDRP14	128	512	1024	2
MDRP20	512	256	1024	512
MDRP21	512	16	64	32
MDRP24, 88	512	128	512	64
MDRP25, 46	512	256	512	128
MDRP30	512	256	2048	64
MDRP31, 75, 100	512	128	1024	64
MDRP32	512	256	512	1024
MDRP33	512	64	128	16
MDRP38	512	32	128	16
MDRP39	512	32	256	32
MDRP41	256	128	512	64
MDRP42, 95	512	256	256	64
MDRP44	512	16	128	64
MDRP45	512	512	256	128
MDRP50	256	256	1024	512
MDRP57	512	256	2048	128
MDRP60, 98	512	32	128	64
MDRP62	512	16	256	256
MDRP63	512	64	128	512
MDRP67	512	32	512	64
MDRP71, 103	512	256	128	512
MDRP72	512	256	512	512
MDRP74	512	512	1024	64
MDRP83	512	512	256	16
MDRP87	512	128	512	128
MDRP92, 94	512	256	512	32
MDRP96	512	256	512	128
MDRP101	512	256	256	32
MDRP105	512	32	128	128
MDRP106	512	64	512	64

MGO, methylglyoxal; IPM, imipenem; AMK, amikacin; CPFX, ciprofloxacin; MIC, minimum inhibitory concentration; MDRP, multidrug-resistant Pseudomonas aeruginosa. MIC determinations were repeated at least three times.

### Susceptibilities of MDRP strains to methylglyoxal

To evaluate the antibacterial activity of methylglyoxal against clinically isolated MDRP strains, we determined MICs using the 53 confirmed MDRP strains. The MIC of methylglyoxal for most of the MDRP strains was 512 μg/ml (Table 2), whereas the susceptibilities of these strains to imipenem, amikacin, and ciprofloxacin were different. The methylglyoxal concentration at which MDRP14 was susceptible was 128 μg/ml and that at which MDRP4, 5, 41, and 50 were susceptible was 256 μg/ml. We also tested the methylglyoxal susceptibility of the drug-sensitive wild-type strain *P. aeruginosa* PAO1. The MIC of methylglyoxal for PAO1 was 512 μg/ml (Table 3), which was the same that for most of the MDRP strains.

**Table 3:** **Susceptibility of drug efflux mutants to antimicrobial compounds**.

	**MIC (μg/ml)**			
**Strain**	**MGO**	**IPM**	**AMK**	**CPFX**
PAO1 (*P. aeruginosa* wild-type)	512	2	16	0.25
PMX52 (Δ*mexAB oprM mexCD oprJ mexEF oprN mexXY mexHI opmD*)	512	2	2	0.016
MG1655 (*E. coli* wild-type)	256	0.25	1	0.031
NKE1329 (Δ*acrB acrD mdtABC mdtEF acrEF*)	256	0.5	1	≤0.0078
NKE95 (Δ*tolC*)	256	0.5	0.5	≤0.0078
ATCC14028s (*S. enterica* wild-type)	256	0.25	4	0.031
NKS196 (Δ*acrAB acrEF acrD mdtABC mdsABC emrAB mdfA mdtK macAB*)	256	0.5	2	≤0.0078
NKS233 (Δ*tolC*)	256	0.5	2	≤0.0078

MGO, methylglyoxal; IPM, imipenem; AMK, amikacin; and CPFX, ciprofloxacin; MIC, minimum inhibitory concentration; MDRP, multidrug-resistant Pseudomonas aeruginosa. MIC determinations were repeated at least three times.

### Effect of drug efflux systems in *P. aeruginosa*, *E. coli*, and *S. enterica* to methylglyoxal

**Figure 1:**
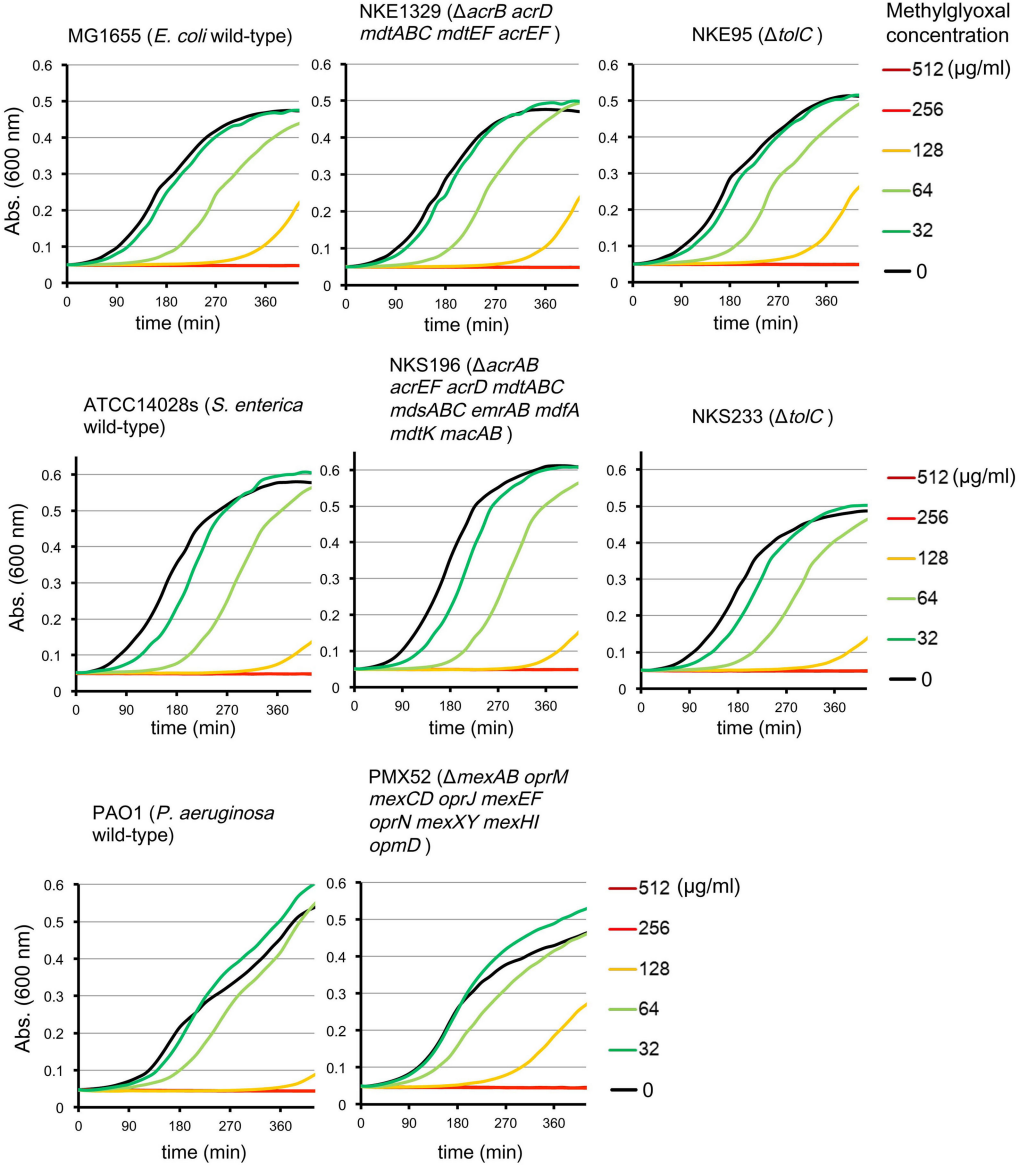
**Effects of methylglyoxal on the growth of *E. coli*, *S. enterica*, and *P. aeruginosa***. Growth of *E. coli* (MG1655, NKE1329, and NKE95), *S. enterica* (ATCC14028s, NKS196, and NKS233), and *P. aeruginosa* (PAO1 and PMX52) strains were measured in liquid medium with or without methylglyoxal.

Multidrug efflux pumps in *P. aeruginosa*, such as MexAB-OprM, MexCD-OprJ, MexEF-OprN, and MexXY, have been shown to be significant determinants of multidrug resistance in laboratory and clinical isolates (Poole, 2004; Piddock, 2006; Lister et al., 2009). The existence of an additional multidrug efflux system, MexHI-OpmD, was also previously reported (Sekiya et al., 2003) in this organism. Because multidrug efflux systems display the ability to transport various structurally unrelated drugs, we investigated whether methylglyoxal is exported by these drug efflux systems in *P. aeruginosa*. For this purpose, we measured MIC of methylglyoxal for the wild-type *P. aeruginosa* strain PAO1 and its efflux-deficient mutant strain PMX52 (Δ*mexAB oprM mexCD oprJ mexEF oprN mexXY mexHI opmD*). Although PMX52 was more susceptible to amikacin and ciprofloxacin than PAO1, MIC of methylglyoxal for these strains was the same. This suggests that methylglyoxal is not recognized by drug efflux systems in *P. aeruginosa*. To confirm whether same phenomenon could be observed in other gram-negative bacteria, we determined MICs of methylglyoxal for the efflux-deficient mutants of *E. coli* and *S. enterica* serovar Typhimurium. There are five RND-type drug efflux systems (AcrAB, AcrD, MdtABC, MdtEF, and AcrEF) in *E. coli*, and all of them require the TolC outer membrane channel for their function (Nishino et al., 2003). To investigate the defect of these drug efflux systems in *E. coli*, we measured MICs of methylglyoxal for MG1655 (wild-type), NKE1329 (Δ*acrB acrD mdtABC mdtEF acrEF*), and NKE95 (Δ*tolC*) strains. The susceptibility of NKE1329 and NKE95 to methylglyoxal was same as that of the wild-type strain, although they were more susceptible to ciprofloxacin than the wild-type strain. *S. enterica* serovar Typhimurium harbors at least nine drug efflux systems belonging to RND, multidrug and toxic compound extrusion, and ATP-binding cassette (ABC) superfamilies (Nishino et al., 2006). Seven of them (AcrAB, AcrEF, AcrD, MdtABC, MdsAbC, EmrAB, and MacAB) require TolC for their function (Horiyama et al., 2010). For *S. enterica*, we used ATCC14028s (wild-type), NKS196 (Δ*acrAB acrEF acrD mdtABC mdsABC emrAB mdfA mdtK macAB*), and NKS233 (Δ*tolC*) strains. Although NKS196 and NKS233 were more sensitive to ciprofloxacin than the wild-type strain ATCC14028s, MICs of methylglyoxal for ATCC14028s, NKS196, and NKS233 were the same. In addition to MIC determination using agar plates, we tested the effect of methylglyoxal on bacterial growth in liquid medium. The growth of *E. coli* (MG1655, NKE1329, and NKE9) and *Salmonella* (ATCC14028s, NKS196, and NKS233) strains was inhibited by methylglyoxal at a concentration of 256 μg/ml, and the growth of *P. aeruginosa* (PAO1 and PMX52) strains was inhibited at 512 μg/ml, which is consistent with MICs determined (Figure 1). These data suggest that methylglyoxal is not recognized by drug efflux systems in *E. coli* or *S. enterica*.

In this study, we showed that methylglyoxal equally inhibits drug-susceptible *P. aeruginosa* and MDRP at concentrations of 128–512 μg/ml (1.7–7.1 mM). Methylglyoxal is a key antimicrobial component of manuka honey, and manuka honey has previously been suggested as a topical treatment option for burn patients infected with *P. aeruginosa* (Cooper et al., 2002a). Jenkins and Cooper reported that MICs of manuka honey for MRSA and methicillin-resistant *P. aeruginosa* were 6–7% w/v (Jenkins and Cooper, 2012). This corresponds to 50–100 μg/ml methylglyoxal when manuka honey contains 7% of methylglyoxal. Cooper et al. also reported that MIC for *E. coli* is 16% w/v (Cooper et al., 2010), which corresponds to approximately 200 μg/ml methylglyoxal. It was previously reported that methylglyoxal is the dominant antibacterial constituent of manuka honey and that MIC of methylglyoxal for *E. coli* and *S. aureus*, determined using the agar well diffusion assay, is 1.1 mM (79.3 μg/ml) (Mavric et al., 2008). Our data showed that methylglyoxal itself inhibits the growth of MDRP strains at high concentrations, suggesting that methylglyoxal activity might be enhanced when in honey solution. Further research is required to demonstrate whether methylglyoxal and manuka honey exert their antibacterial effects through a common mechanism. We also showed that methylglyoxal is not recognized by drug efflux systems in *P. aeruginosa*, *E. coli*, and *S. enterica*.

### Conflict of interest statement

The authors declare that the research was conducted in the absence of any commercial or financial relationships that could be construed as a potential conflict of interest.
